# Mathematical modelling and application of frog choruses as an autonomous distributed communication system

**DOI:** 10.1098/rsos.181117

**Published:** 2019-01-09

**Authors:** Ikkyu Aihara, Daichi Kominami, Yasuharu Hirano, Masayuki Murata

**Affiliations:** 1 Graduate School of Systems and Information Engineering, University of Tsukuba, Ibaraki 305-8573, Japan; 2 Graduate School of Economics, Osaka University, Osaka 560-0043, Japan; 3 Graduate School of Information Science and Technology, Osaka University, Osaka 565-0871, Japan

**Keywords:** nonlinear dynamics, biomimetics, acoustic communication, frog algorithm

## Abstract

Interactions using various sensory cues produce sophisticated behaviour in animal swarms, e.g. the foraging behaviour of ants and the flocking of birds and fish. Here, we investigate the behavioural mechanisms of frog choruses from the viewpoints of mathematical modelling and its application. Empirical data on male Japanese tree frogs demonstrate that (1) neighbouring male frogs avoid call overlaps with each other over a short time scale and (2) they collectively switch between the calling state and the silent state over a long time scale. To reproduce these features, we propose a mathematical model in which separate dynamical models spontaneously switch due to a stochastic process depending on the internal dynamics of respective frogs and also the interactions among the frogs. Next, the mathematical model is applied to the control of a wireless sensor network in which multiple sensor nodes send a data packet towards their neighbours so as to deliver the packet to a gateway node by multi-hop communication. Numerical simulation demonstrates that (1) neighbouring nodes can avoid a packet collision over a short time scale by alternating the timing of data transmission and (2) all the nodes collectively switch their states over a long time scale, establishing high network connectivity while reducing network power consumption. Consequently, this study highlights the unique dynamics of frog choruses over multiple time scales and also provides a novel bio-inspired technology that is applicable to the control of a wireless sensor network.

## 1. Introduction

Animals exhibit various types of collective behaviour in the form of swarms. For instance, ants forage for food resources by cooperating with other individuals [1]; birds and fish form a flexible flocking as a result of the interactions among them [2,3]. In these systems, each animal dynamically controls its motion by interacting with its neighbours using various sensory cues, which results in high performance of the swarms. It is known that experimental and theoretical studies of animal swarms contribute to further understanding of the interaction mechanisms among multiple animals [4]. On the other hand, the sophisticated behaviour of animals has inspired the development of a novel technology called *swarm intelligence*. Various theoretical studies show that swarm intelligence (e.g. ant-colony optimization and particle-swarm optimization) can solve real-world problems, especially in the fields of information and communication technologies (ICT) [5]. However, the studies of swarm intelligence are currently based on the behaviour of just a few species of animals. Theoretical studies bridging various animal behaviours and real-world problems would contribute to further development of swarm intelligence in the field of ICT.

This study aims to examine the mechanisms inherent in the acoustic communication of frogs. It is well known that various animals use sounds for the purpose of communication. For example, human conversation is based on sounds; whales can interact with each other by using low-frequency sounds even when they are far apart [6]; male birds, frogs and insects produce sounds to attract conspecific females [7–9]. It is essential for these animals to alternate the timing of their acoustic signals because the temporal overlap of the signals masks the information included in their own signals. In particular, frogs are abundant but unique animals that exhibit sophisticated behaviour in their acoustic communication. In general, a male frog produces successive calls in order to attract conspecific females and claim his own territories to other male frogs [8,9]. The important point is that many male frogs call at the same breeding site, making it very difficult for them to alternate call timings with each other. Experimental studies have reported that various species of frogs can avoid call overlaps with neighbours over a short time scale despite their complicated acoustic environment [8–14]. In addition, male frogs can synchronize the start and stop of their calling behaviour over a longer time scale, resulting in the formation of collective choruses [8,9,15,16]. Subsequently, male frogs can take a rest at each interval between the choruses, allowing them to reduce both energy consumption and physical fatigue.

The essence of frog choruses described above is that neighbouring frogs alternate their calls in a local population while forming collective choruses as a whole system. Such behaviour including both alternating and collective features is applicable to the control of a wireless sensor network. A wireless sensor network is a distributed communication system using many sensor nodes that transmit a data packet with their neighbours so as to deliver the packet to a gateway node by multi-hop communication [17,18]. This technique is quite promising for the future Internet of Things (IoT) and cyber physical systems that are indispensable for the safety and security of human lives. In those systems, the sensor nodes are deployed over a wide region, which allows users to collect various types of geographically dispersed information such as the spatial distribution of climatic factors and household power consumption [18]. For robust and continuous data collection, a wireless sensor network is required to solve the following problems: (1) neighbouring nodes need to alternate the timings of data transmission to avoid a packet collision and (2) the nodes need to establish high connectivity for multi-hop communication while reducing their power consumption over a long time scale. This study aims to solve these problems by using a mathematical model of frog choruses.

In this study, we first propose a mathematical model that describes the collective and alternating features of frog choruses over multiple time scales (§2), and then demonstrate that the proposed model can solve the problems inherent in the control of a wireless sensor network (§3). It should be noted that Mutazono *et al.* carried out a seminal work on the application of frogs’ behaviour to the control of a wireless sensor network [19]. In that study, they focused on the alternating chorus pattern of male Japanese tree frogs that Aihara *et al.* had found and modelled by using a phase oscillator model [20,21], and also focused on the satellite behaviour of male frogs. The satellite behaviour describes the strategy that male frogs stay silent in the vicinity of a calling male for a long time so as to intercept a female that is attracted to the calling male [8]. Thus, the satellite behaviour is based on *an inhibitory interaction* among male frogs that inhibits the calling behaviour of neighbouring males and then allows them to reduce energy consumption. Mutazono *et al.* showed that the satellite behaviour is useful for reducing the power consumption in a wireless sensor network while the alternating chorus pattern is useful for avoiding a packet collision among neighbouring nodes. By contrast, this study focuses on *an excitatory interaction* among male frogs that activate the calling behaviour of neighbouring males and then reproduces their collective choruses over a long time scale. This feature over a long time scale is a novel point of our study compared to the study by Mutazono *et al.* [19].

## 2. Frog choruses

In this section, we first explain the dominant features of frog choruses on the basis of empirical data (§2.1). Then, we propose a mathematical model of frog choruses (§2.2) and examine the validity of the proposed model by comparing the results of numerical simulations with the empirical data (§§2.3 and 2.4).

### 2.1. Empirical data

**Figure 1.**
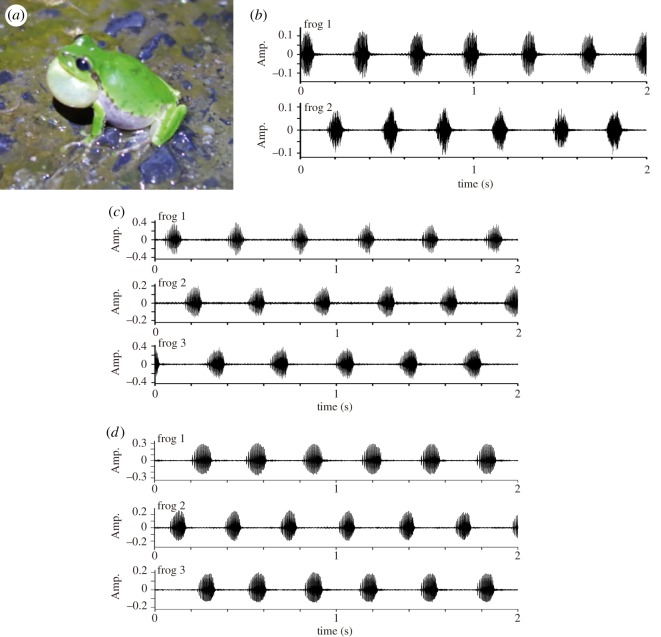
Empirical data on the choruses of male Japanese tree frogs over a short time scale. (*a*) Photograph of a male Japanese tree frog. (*b*) Anti-phase synchronization of two frogs. (*c*) Tri-phase synchronization of three frogs. (*d*) Clustered anti-phase synchronization of three frogs. The male frogs tend to avoid call overlaps with each other over a short time scale. The data shown here are obtained from our previous study [12].

Here, we explain the dominant features of frog choruses on the basis of empirical data on male Japanese tree frogs (figure 1*a*) that were obtained from our previous study of [12]. Japanese tree frogs (*Hyla japonica*) are widely distributed in Japan from Kagoshima prefecture in the southwest to Hokkaido in the northeast. It is observed that many male frogs chorus at the same breeding site such as a paddy field with shallow water [22]. To investigate their behaviour, we performed indoor experiments [12]. In each experiment, three male frogs were put into small mesh cages, respectively. The cages were set along a straight line at intervals of 50 cm, and the calling behaviour of male frogs was recorded by three microphones that were placed in the vicinity of each cage. We analysed the audio data of 4 h according to the method of independent component analysis, and separated the call signals of respective frogs. These experiments and analyses were carried out on four datasets with 12 male frogs in total [12].

In the previous study [12], we reported that male Japanese tree frogs exhibit various types of alternating chorus patterns. Figure 1*b* shows the separated call signals of two frogs that were obtained from the previous study. It is demonstrated that they call alternately with each other, which can be understood as the anti-phase synchronization of two frogs [13,21,23]. In addition, figure 1*c*,*d* shows the separated call signals of three frogs. It is demonstrated that three frogs call in turns (figure 1*c*) or one frog synchronizes in anti-phase with the remaining two frogs that synchronize in in-phase (figure 1*d*), which can be understood as the tri-phase synchronization of three frogs and the clustered anti-phase synchronization of three frogs, respectively [12]. These alternating patterns allow male frogs to avoid call overlaps as often as possible [12,13], and then they would effectively advertise themselves to conspecific females [12,13,24].

**Figure 2.**
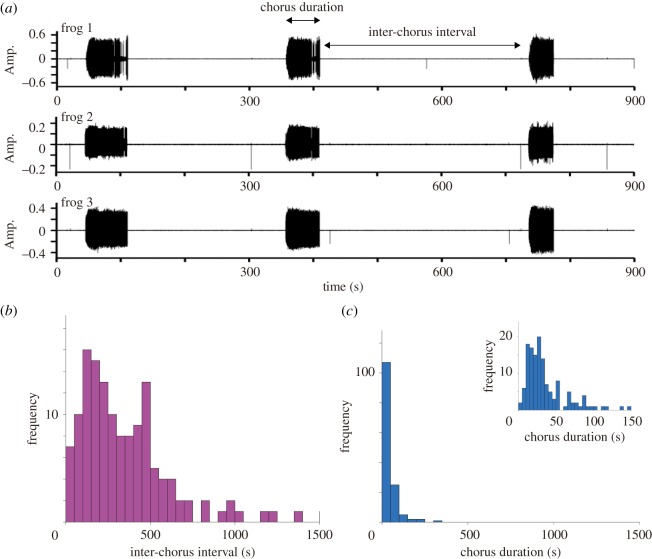
Empirical data on the choruses of male Japanese tree frogs over a long time scale. (*a*) Collective transitions between the calling state and the silent state. Over a long time scale, the male frogs almost synchronize the transitions between the calling state and the silent state with each other, resulting in the formation of the collective choruses. (*b*) Histogram of the inter-chorus interval. (*c*) Histogram of the chorus duration. The inset of figure 2*c* shows the enlarged histogram of the chorus duration.

In this study, we newly analyse the properties of frog choruses over a longer time scale. Prior to the analysis, we carefully checked the quality of the sound-source separation and excluded just three choruses in which the separation did not work well, from 143 choruses. Figure 2*a* shows the separated call signals of three frogs over 15 min. It is demonstrated that they almost synchronize the start and stop of their calling behaviour, resulting in the formation of collective and intermittent choruses. In this study, we define *a calling state* as the period in which a male frog produces successive calls continuously, and define *a silent state* as the period in which a male frog stays silent without producing any call. In addition, we analyse the detailed properties of the transition between the calling state and the silent state. The period of a calling state is first estimated for respective frogs according to the method explained in our previous studies [25,26]; *a chorus* of male frogs is then detected as a period that includes a partial overlap of calling states among the three frogs. Next, we determine the inter-chorus interval and chorus duration as displayed in figure 2*a*, and calculate those values from the four datasets of 12 frogs. Figure 2*b*,*c* shows the histogram of the inter-chorus interval and that of the chorus duration, respectively. This analysis indicates that the histogram of the inter-chorus interval varies considerably from tens of seconds to hundreds of seconds, and also demonstrates that the inter-chorus interval is relatively longer than the chorus duration.

Thus, the empirical data on male Japanese tree frogs reveals the alternating chorus patterns over a short time scale, and the collective transition between the calling state and the silent state over a long time scale. It should be noted that these features are consistent with the observations on various species of frogs [8–16].

### 2.2. Mathematical modelling

Here, we propose a novel mathematical model that describes the dominant features of frog choruses (i.e. the avoidance of call overlaps over a short time scale and the collective transition between the calling state and the silent state over a long time scale). Our previous studies [12,13,23,27] have shown that the avoidance of call overlaps over a short time scale can be reproduced as the stable equilibrium states of a phase-oscillator model [28] that follows a deterministic process. By contrast, our empirical data indicate that the inter-chorus interval varies considerably from tens of seconds to hundreds of seconds over a long time scale (figure 2*b*). We consider that such a variation of the transition over a long time scale can be modelled as a stochastic process and not as a deterministic process. Hence, we model the mechanisms of frog choruses as a hybrid system in which separate deterministic models including an extended version of a phase oscillator model are switched by a stochastic process associated with the internal dynamics of respective frogs as well as the interactions among them.

In the mathematical model, we use four variables: a state *s*_*n*_, a phase *θ*_*n*_, physical fatigue *T*_*n*_ and energy *E*_*n*_. Here, we explain the definitions of these variables with reference to our empirical data and related studies.

— *State*: Our empirical data show that male Japanese tree frogs collectively switch between the calling state and the silent state (figure 2*a*). Here, we distinguish between the calling state and the silent state by using an integer *s*_*n*_; it is assumed that *s*_*n*_ = 0 represents the silent state of the *n*th frog, and *s*_*n*_ = 1 represents the calling state of the *n*th frog. We then model the collective transition by considering the excitatory acoustic interaction among male frogs that activate the calling behaviour of neighbouring frogs (equation (2.12)), which is necessary for the occurrence of the collective transition.
— *Phase*: Our empirical data show that a male frog produces successive calls continuously during the calling state (figure 1*b–d*). To reproduce this periodic feature, we define a phase *θ*_*n*_ ∈ [0, 2*π*) (mod 2*π*) for respective frogs; it is assumed that the *n*th frog produces a call at *θ*_*n*_ = 0 [12,13,27]. We use this variable to propose a deterministic model in which *θ*_*n*_ repeatedly increases from 0 to 2*π* during the calling state (equation (2.1)) and does not change during the silent state (equation (2.4)).
— *Physical fatigue*: To produce successive calls, a male frog must inflate and deflate his large vocal sac at a high repetition rate [8,9]. Such vigorous movement is likely to cause severe physical fatigue in male frogs. To describe this feature, we define physical fatigue *T*_*n*_ ∈ [0, *T*_max_] for respective frogs; it is assumed that the physical fatigue *T*_*n*_ increases during the calling state (equation (2.2)), and decreases during the silent state (equation (2.5)). We then propose a stochastic model in which the amount of physical fatigue affects the probability of the transition between the calling state and the silent state (equations (2.8) and (2.10)).
— *Energy*: Various experimental studies have reported that a male frog loses a large amount of his weight when joining choruses at night [8,29–31]. This indicates that the calling behaviour of a male frog causes not only physical fatigue but also severe energy consumption. To model this feature, we define energy *E*_*n*_ ∈ [0, *E*_max_] for respective frogs; it is assumed that the energy *E*_*n*_ takes the maximum value *E*_max_ at an initial condition, and then decreases during the calling state (equation (2.3)) while remaining constant during the silent state (equation (2.6)). We use this variable to propose a stochastic model in which the amount of energy affects the probability of the transition between the calling state and the silent state (equation (2.11)).

On the basis of the above variables (i.e. the state *s*_*n*_, the phase *θ*_*n*_, the physical fatigue *T*_*n*_ and the energy *E*_*n*_), we describe the calling state and the silent state of respective frogs as separate deterministic models.

First, the calling state of the *n*th frog (*s*_*n*_ = 1 for *n* = 1, 2, …, *N*) is modelled as follows:

2.1 $$\frac{\text{d}\theta_{n}}{\text{d}t}=\omega +\sum_{m\;\mathrm{for}\;s_{m}=1\;\mathrm{and}\;r_{nm}<r_{0}}^{N}\delta (\theta_{m})\Gamma_{nm}(\theta_{n}-\theta_{m}),$$

2.2 $$\frac{\text{d}T_{n}}{\text{d}t}=\delta (\theta_{n})$$

2.3 $$\;\mathrm{and}\;\frac{\text{d}E_{n}}{\text{d}t}=-\delta (\theta_{n}).$$

Equation (2.1) is based on a phase oscillator model [28]. Here, *ω* is a positive parameter that represents the intrinsic angular velocity of a male frog (in other words, 2*π*/*ω* represents the intrinsic inter-call interval of a male frog) [12,13,23,27]. The second term on the right-hand side of equation (2.1) describes the instantaneous interaction among neighbouring frogs. In this term, *r*_*nm*_ is the distance between the *n*th and *m*th frogs; *r*_0_ is a threshold within which male frogs can interact with each other; *Γ*_*nm*_(*θ*_*n*_ − *θ*_*m*_) is a 2*π*-periodic function of the phase difference *θ*_*n*_ − *θ*_*m*_ ∈ [0, 2*π*) (mod 2*π*) that represents the interaction between the *n*th and *m*th frogs [12,13,23,27]. In addition, *δ*(*θ*_*m*_) is a delta function that describes the instantaneous interaction. Here, we assume that *δ*(*θ*_*m*_) = ∞ at *θ*_*m*_ = 0 and *δ*(*θ*_*m*_) = 0 otherwise. Then, we also assume that *δ*(*θ*_*m*_) satisfies the relationship $\int_{t_{m,i}-\epsilon }^{t_{m,i}+\epsilon }\delta (\theta_{m}(t))\;\text{d}t=1$ for each call timing *t*_*m*,*i*_ at which the phase hits 0. (Here, *i* represents the index of calls produced by the *m*th frog, and *ε* is a positive parameter that is much smaller than an inter-call interval.) It should be noted that, because of this assumption $\int_{t_{m,i}-\epsilon }^{t_{m,i}+\epsilon }\delta (\theta_{m}(t))\;\text{d}t=1$, the delta function assumed here is different from the Dirac delta function. Consequently, the second term on the right-hand side of equation (2.1) denotes that the *n*th frog instantaneously interacts with the *m*th frog that is calling (*s*_*m*_ = 1) and is positioned within their interaction range (*r*_*nm*_ < *r*_0_). Equations (2.2) and (2.3) describe the dynamics of the physical fatigue *T*_*n*_ and the energy *E*_*n*_ by the delta function *δ*(*θ*_*n*_). These time differential equations mean that the physical fatigue *T*_*n*_ is incremented by 1 and the energy *E*_*n*_ is decremented by 1 every time the phase *θ*_*n*_ hits 0 (namely, every time the *n*th frog produces a call); this is because the delta function assumed here gives the deviation of *T*_*n*_ and *E*_*n*_ at each call timing *t*_*n*,*j*_ as $\Delta T_{n}=\int_{t_{n,j}-\epsilon }^{t_{n,j}+\epsilon }(\text{d}T_{n}/\text{d}t)\;\text{d}t=\int_{t_{n,j}-\epsilon }^{t_{n,j}+\epsilon }\delta (\theta_{n}(t))\;\text{d}t=1$ and $\Delta E_{n}=\int_{t_{n,j}-\epsilon }^{t_{n,j}+\epsilon }(\text{d}E_{n}/\text{d}t)\;\text{d}t=\int_{t_{n,j}-\epsilon }^{t_{n,j}+\epsilon }(-\delta (\theta_{n}(t)))\;\text{d}t=-1$. (Here, *j* represents the index of calls produced by the *n*th frog.)

The silent state of the *n*th frog (*s*_*n*_ = 0 for *n* = 1, 2, …, *N*) is modelled as follows:

2.4 $$\frac{\text{d}\theta_{n}}{\text{d}t}=0,$$

2.5 $$\frac{\text{d}T_{n}}{\text{d}t}=-\alpha$$

2.6 $$\;\mathrm{and}\;\frac{\text{d}E_{n}}{\text{d}t}=0.\;$$

In this study, we define the silent state as the period in which a male frog stays silent without producing any call. In equation (2.4), the phase *θ*_*n*_ does not change and remains constant, which is consistent with our definition of the silent state. Equations (2.5) and (2.6) describe the dynamics of the physical fatigue and the energy during the silent state, meaning that the physical fatigue *T*_*n*_ decreases at a constant rate of *α* (equation (2.5)) and the energy *E*_*n*_ does not change during the silent state (equation (2.6)).

Next, we model the transition between the calling state and the silent state as a Markov process in which male frogs determine the occurrence of the transition depending on their current conditions. The probability of the transition from the calling state (*s*_*n*_ = 1) to the silent state (*s*_*n*_ = 0) is modelled as follows:

2.7 $$P_{n}^{\mathrm{call}\to \mathrm{silent}}=G_{1}(T_{n}),$$

2.8 $$G_{1}(T_{n})=\frac{1}{\exp(-\gamma (T_{n}-\Delta T))+1}.$$

**Figure 3.**
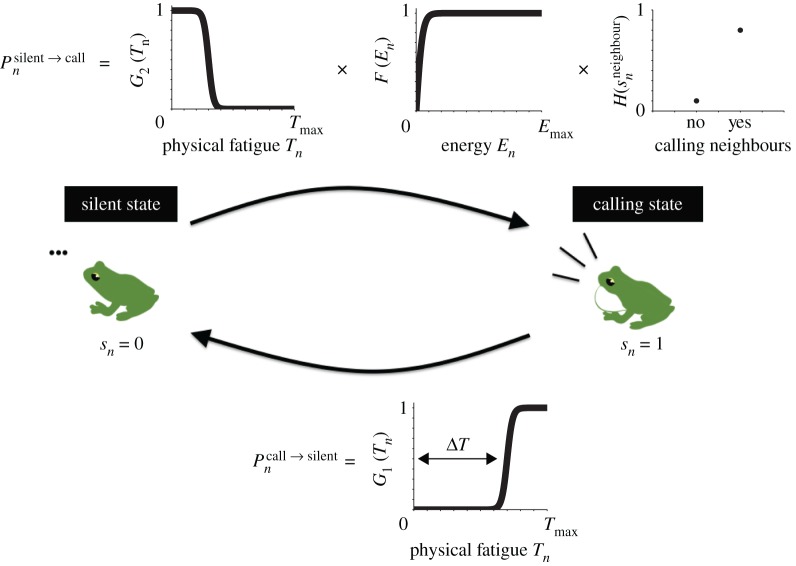
Schematic diagram of our mathematical model on frog choruses. The transition between the calling state and the silent state is modelled as a Markov process in which the probability of the transition is described by the current condition of respective frogs as well as by the interactions among neighbouring frogs.

Here, we use a logistic function with two positive parameters *γ* and Δ*T*. While *γ* represents the steepness of this function, Δ*T* represents the inflection point of this function. Note that Δ*T* approximately gives the number of calls produced in each section of the calling state. A representative shape of this function is shown in figure 3, demonstrating that a male frog with a larger amount of physical fatigue transits from the calling state to the silent state with higher probability.

The probability of the transition from the silent state (*s*_*n*_ = 0) to the calling state (*s*_*n*_ = 1) is modelled as follows:

2.9 $$P_{n}^{\mathrm{silent}\to \mathrm{call}}=G_{2}(T_{n})F(E_{n})H(s_{n}^{\mathrm{neighbour}}),$$

where

2.10 $$G_{2}(T_{n})=\frac{1}{\exp(\gamma (T_{n}-(T_{\max}-\Delta T)))+1},$$

2.11 $$F(E_{n})=-\frac{2}{\exp(\beta E_{n})+1}+1$$

and

2.12 $$H(s_{n}^{\mathrm{neighbour}})=\left\{ \begin{array}{l} p_{\mathrm{high}}\left(\mathrm{If}\;a\;\mathrm{vector}\;s_{n}^{\;\mathrm{neighbour}}\;\mathrm{has}\;\mathrm{one}\;\mathrm{or}\;\mathrm{more}\;\mathrm{elements}\;\mathrm{of}\;1\right),\\ p_{\mathrm{low}}\left(\mathrm{If}\;a\;\mathrm{vector}\;s_{n}^{\;\mathrm{neighbour}}\;\mathrm{has}\;\mathrm{no}\;\mathrm{element}\;\mathrm{of}\;1\right). \end{array}\right.$$

Given that the calling behaviour of male frogs causes severe physical fatigue and energy consumption once it starts, this transition requires careful decision making by the male frogs. Hence, we consider the effects of the energy *E*_*n*_ and the states of neighbouring frogs $s_{n}^{\mathrm{neighbour}}$ in addition to the physical fatigue *T*_*n*_. In equation (2.10), we use a logistic function with two positive parameters *γ* and Δ*T* that are the same as in equation (2.8); this is because both equations describe the effect of the physical fatigue *T*_*n*_ on the transition between the calling state and the silent state. In equation (2.11), we also use a logistic function with a different positive parameter *β* that represents the steepness of this function *F*(*E*_*n*_). Consequently, equations (2.10) and (2.11) imply that a male frog with less physical fatigue and more energy transits from the silent state to the calling state with higher probability. As for the excitatory acoustic interaction among male frogs, we assume equation (2.12) in which a male frog determines the probability of the transition depending on the current states of neighbouring frogs. In this equation, a vector $s_{n}^{\mathrm{neighbour}}$ consists of the states of neighbouring frogs that are positioned within their interaction range (i.e. *r*_*nm*_ < *r*_0_); the parameters *p*_high_ and *p*_low_ are assumed to be positive and satisfy the relationship *p*_low_ < *p*_high_ ≤ 1. Consequently, equation (2.12) denotes that the calls of male frogs activate the calling behaviour of neighbouring frogs. The representative shapes of the functions *G*_2_(*T*_*n*_), *F*(*E*_*n*_) and $H(s_{n}^{\mathrm{neighbour}})$ are shown in figure 3.

### 2.3. Parameter values

Here, we fix the parameters of the proposed model (i.e. the deterministic model of the calling state (equations (2.1)–(2.3)), the deterministic model of the silent state (equations (2.4)–(2.6)), the stochastic model of the transition from the calling state to the silent state (equations (2.7) and (2.8)) and the stochastic model of the transition from the silent state to the calling state (equations (2.9)–(2.12))) on the basis of empirical data and speculations on the behaviour of male Japanese tree frogs.

— *Deterministic models*: Our empirical data show that male Japanese tree frogs produce successive calls at a specific interval while avoiding call overlaps with each other (figure 1*b*–*d*). To reproduce this feature, we use a phase oscillator model of equation (2.1) in which 2*π*/*ω* represents the inter-call interval of each frog. From our empirical data explained in §2.1, we estimate this interval for respective frogs and confirm that the median value of the interval is 0.306 s. On the basis of this result, we fix the parameter as 2*π*/*ω* = 0.306 s. In addition, our previous study on a phase oscillator model shows that a sinusoidal interaction term with the first-order component and the second-order component of a Fourier series can reproduce the alternating chorus patterns of male frogs (e.g. anti-phase synchronization of two frogs, tri-phase synchronization of three frogs, and clustered anti-phase synchronization of three frogs) as stable equilibrium states [12]. Therefore, we assume the same interaction term as follows:
  2.13 $$\Gamma_{nm}(\theta_{n}-\theta_{m})=K_{nm}[\sin(\theta_{n}-\theta_{m})-k\sin(2(\theta_{n}-\theta_{m}))].$$
  In our experiments, three male frogs were deployed at intervals of 50 cm [12]. In our experience, this is a sufficiently short distance for each frog to hear the calls of other frogs. Therefore, we assume that the condition *r*_*nm*_ < *r*_0_ of equation (2.1) always holds for all the pairs of frogs. Moreover, the coupling coefficients among neighbouring frogs are fixed as *K*_*nm*_ = 0.20 and *k* = 0.18 based on our previous study [12] so as to reproduce the tri-phase synchronization of three frogs and also the clustered anti-phase synchronization of three frogs as bistable equilibrium states. As for the properties of frog choruses over a long time scale, our empirical data demonstrates that the inter-chorus interval is longer than the chorus duration (figure 2*b*,*c*), indicating that each frog stays silent for a sufficient amount of time and then starts calling. On the basis of this feature, we fix the recovery rate of the physical fatigue during the silent state as *α* = 0.12 × *ω*/2*π* that is much less than the increase rate of the physical fatigue during the calling state that is approximately given by *ω*/2*π*.
— *Effect of physical fatigue in stochastic models*. Our model assumes that the physical fatigue *T*_*n*_ affects the probability of the transition between the calling state and the silent state. In particular, the transition from the calling state to the silent state is modelled by the logistic function of equation (2.8) in which the positive parameter Δ*T* approximately gives the number of calls produced in each section of the calling state. To fix this parameter, we estimate the duration of each section of the calling state from our empirical data and confirm that the median value of the duration is 25.53 s. Combined with the fact that *ω*/2*π* represents the number of calls produced per second, we fix the parameter as Δ*T* = 25.53 × *ω*/2*π*. Accordingly, we fix the maximum physical fatigue as *T*_max_ = 1.2 × Δ*T* because *T*_max_ should be slightly larger than Δ*T* as explained in figure 3. Moreover, we fix the steepness of the logistic functions (equations (2.8) and (2.10)) as *γ* = 0.5 in order to gradually change these functions between 0 and *T*_max_.
— *Effect of energy in stochastic models*. From our experiments on male Japanese tree frogs, we obtained the audio data of 4 h that was recorded at night (§2.1 and [12]). In our experience, these data almost cover the period in which male Japanese tree frogs chorus at night. Hence, we regard the total number of calls produced by respective frogs during this period as the maximum energy *E*_max_. We analyse the audio data of 4 h with 12 frogs, and confirm that the median value of the total number of calls is 3878. Based on this result, we fix the maximum energy as *E*_max_ = 3878. On the other hand, our model assumes that the energy of each frog affects the probability of the transition from the silent state to the calling state according to the logistic function (equation (2.11)). To gradually change this function between 0 and *E*_max_, we fix the steepness of the function as *β* = 0.01.
— *Effect of interactions in stochastic models*. Our empirical data show that male Japanese tree frogs collectively switch between the calling state and the silent state (figure 2*a*). In general, such a collective transition requires the excitatory interaction among male frogs. In our model, the interaction is described by the function $H(s_{n}^{\mathrm{neighbour}})$ of equation (2.12) that includes two parameters *p*_high_ and *p*_low_. We fix these parameters as *p*_high_ = 0.80 and *p*_low_ = 0.01 so as to satisfy their constraint *p*_low_ < *p*_high_ ≤ 1.

To perform numerical simulation, we treat the transition between the silent state and the calling state as a discrete-time Markov process. Namely, we discretely determine the occurrence of the transition according to $P_{n}^{\mathrm{call}\to \mathrm{silent}}$ or $P_{n}^{\mathrm{silent}\to \mathrm{call}}$ at a specific interval Δ*t*_update_. Given that *E*_*n*_ and *T*_*n*_ change at the inter-call interval of 2*π*/*ω* during the calling state, we fix this parameter as Δ*t*_update_ = 3.5 s, which is much longer than 2*π*/*ω* = 0.306 s. It should be noted that we cannot treat the transition as a continuous-time Markov process in this study because the physical fatigue *T*_*n*_, for instance, changes discretely in time during the calling state (equation (2.2)) and therefore the probability $P_{n}^{\mathrm{call}\to \mathrm{silent}}$ given by *T*_*n*_ (equations (2.7) and (2.8)) is also discontinuous in time.

### 2.4. Numerical simulations

To confirm the validity of the proposed model, we perform numerical simulation under the assumption of equations (2.1)–(2.12) with the parameter values that are fixed as explained in §2.3. With respect to the initial conditions of the simulation, the state *s*_*n*_, the physical fatigue *T*_*n*_ and the phase *θ*_*n*_ are randomized while the energy *E*_*n*_ is fixed as the maximum value *E*_max_. In addition, the phase *θ*_*n*_ is randomized every time the *n*th frog switches its state from the silent state to the calling state.

**Figure 4.**
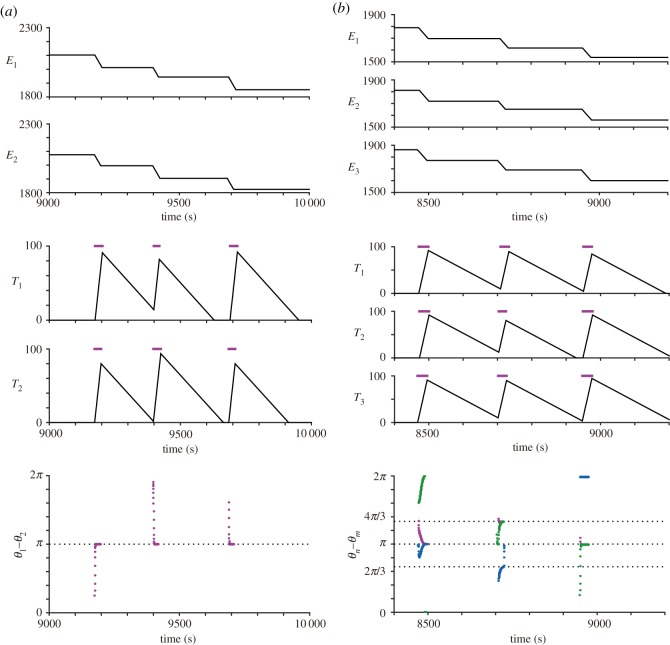
Numerical simulations of our mathematical model on the dynamics of the energy *E*_*n*_, the physical fatigue *T*_*n*_, and the phase difference *θ*_*n*_ − *θ*_*m*_. (*a*) The simulation of two frogs. (*b*) The simulation of three frogs. These results reproduce the collective transition between the calling state and the silent state over a long time scale as well as the alternating chorus patterns over a short time scale (i.e. anti-phase synchronization of two frogs, tri-phase synchronization of three frogs and clustered anti-phase synchronization of three frogs). In the bottom panels, pink dots, blue dots and green dots represent the time-series data of *θ*_1_ − *θ*_2_, *θ*_1_ − *θ*_3_ and *θ*_2_ − *θ*_3_, respectively.

Figure 4*a*,*b* shows the results of the simulation on two frogs and three frogs, respectively. The top and middle panels represent the time-series data of the energy *E*_*n*_ and the physical fatigue *T*_*n*_, respectively. The simulation shows that the drop in *E*_*n*_ is almost synchronized among the frogs (top panels), and the increase and decrease in *T*_*n*_ are also synchronized among the frogs (middle panels). This result demonstrates the occurrence of the collective transition between the calling state and the silent state. The bottom panels represent the time series data of the phase difference *θ*_*n*_ − *θ*_*m*_. The simulation shows that the phase difference converges to *π* in the case of two frogs, and converges to a set of (0, *π*) or a set of (2*π*/3, 4*π*/3) in the case of three frogs (see also electronic supplementary material, figure S1A and S1B); these states correspond to anti-phase synchronization of two frogs, clustered anti-phase synchronization of three frogs and tri-phase synchronization of three frogs, respectively.

**Figure 5.**
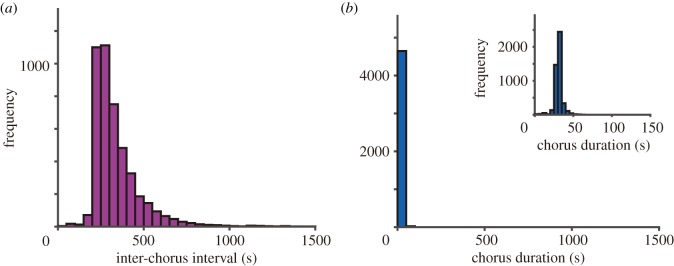
Numerical simulations of our mathematical model on the collective transition between the calling state and the silent state. (*a*) Histogram of the inter-chorus interval. (*b*) Histogram of the chorus duration. It is demonstrated that the inter-chorus interval is longer than the chorus duration, which is consistent with our empirical data of figure 2*b*,*c*.

As for the collective transition, our empirical data indicate that the inter-chorus interval is longer than the chorus duration (figure 2*b*,*c*). To examine whether this feature is reproduced by our mathematical model, we perform further simulation; we calculate the inter-chorus interval and the chorus duration by repeating the same simulation with three frogs 100 times using different initial conditions. The simulation demonstrates that the inter-chorus interval is longer than the chorus duration (figure 5), which is consistent with our empirical data.

## 3. Application to a wireless sensor network

**Figure 6.**
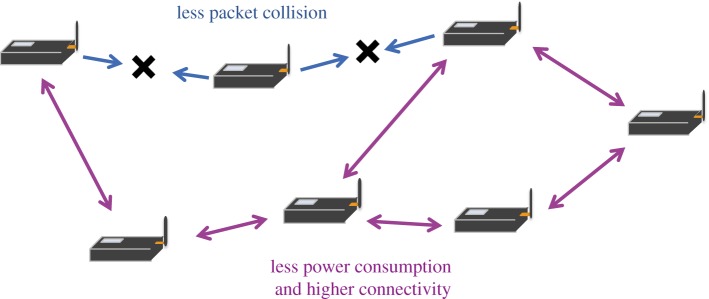
Schematic diagram of a wireless sensor network. A wireless sensor network is a distributed communication system in which many sensor nodes send a data packet towards their neighbours so as to deliver the packet to a gateway node by multi-hop communication. This study aims to decrease the packet collision and power consumption, and increase connectivity in a wireless sensor network by applying our mathematical model inspired by the mechanism of frog choruses.

In the previous section, we proposed a mathematical model of frog choruses and confirmed its validity by comparing the results of numerical simulation with empirical data. Here, we consider the application of the model of frog choruses to an autonomous distributed control of a wireless sensor network (figure 6). In general, a wireless sensor network consists of many sensor nodes that send a data packet towards their neighbours so as to deliver the packet to a gateway node by multi-hop communication [17,18]. In such a communication system with many nodes, it is required that (1) the nodes should avoid a packet collision with their neighbours over a short time scale and (2) the nodes should establish high connectivity for multi-hop communication while reducing their power consumption over a long time scale. We accomplish these requirements by applying the mathematical model of frog choruses that produces alternating patterns over a short time scale and a collective transition over a long time scale.

In this section, we first introduce the mathematical model of a wireless sensor network (§3.1). Then, we fix the parameters of the model by considering an actual situation of a wireless sensor network (§3.2) and perform numerical simulation to confirm the efficacy of the proposed model (§3.3).

### 3.1. Mathematical modelling

Here, we explain how to apply the model of frog choruses to the control of a wireless sensor network.

— *States*: To prolong the lifetime of a wireless sensor network, each node needs to reduce its power consumption as much as possible [17,18]. For this purpose, adaptive switching between *a sleep state* and *an active state* is promising. Here, we define the sleep state as the period in which a sensor node does not send any data packet in order to reduce its power consumption, and define the active state as the period in which a sensor node periodically sends a data packet towards its neighbours. To distinguish between these two states, we use an integer *s*_*n*_; the sleep state of the *n*th node is described as *s*_*n*_ = 0, and the active state is described as *s*_*n*_ = 1. Then, we assume that the nodes in the active state can activate neighbouring sleep nodes in the same way as with frog choruses according to equation (2.12).
— *Transmission interval*: During the active state, each node periodically sends a data packet towards its neighbours. To reproduce this periodic feature, we use the phase *θ*_*n*_; it is assumed that the *n*th node sends a data packet to neighbouring nodes at *θ*_*n*_ = 0. Because a sensor node instantaneously interacts with other nodes when receiving a data packet, we assume the same equation (equation (2.1)) that governs the instantaneous interaction among male frogs. Then, we assume that the phase stays constant during the sleep state in the same way as with frog choruses (equation (2.4)).
— *Duty cycle*: To determine the ratio of the active state and the sleep state, we use the variable *T*_*n*_. It should be noted that this ratio is known as *a duty cycle* in the context of a wireless sensor network [17,18]. In the model of frog choruses, the variable *T*_*n*_ affects the transition from the calling state to the silent state (equation (2.8)) as well as the transition from the silent state to the calling state (equation (2.10)). Thus, *T*_*n*_ is an important factor that determines the time scale of both transitions. For application to a wireless sensor network, we assume that *T*_*n*_ affects the transition between the active state and the sleep state in accordance with the same equations that govern frog choruses (equations (2.8) and (2.10)) so as to introduce a specific time scale for the transition.
— *Battery*: In general, a sensor node is powered by a limited battery that cannot be recharged [17,18]. Here, we use a variable *E*_*n*_ to describe the battery of each node; it is assumed that the battery *E*_*n*_ decreases during the active state (equation (2.3)) and stays constant during the sleep state (equation (2.6)). For practical use, it can be a problem that some nodes consume batteries more quickly than other nodes; this is because excessive power consumption of specific nodes can drastically reduce network connectivity [32,33]. To avoid this problem by employing the model of frog choruses, we assume that the battery *E*_*n*_ affects the probability of the transition from the sleep state to the active state in accordance with equation (2.11).

### 3.2. Parameter values

To perform numerical simulation, we fix the parameters of our model (i.e. the deterministic model of the active state (equations (2.1)–(2.3)), the deterministic model of the sleep state (equations (2.4)–(2.6)), the stochastic model of the transition from the active state to the sleep state (equations (2.7) and (2.8)), and the stochastic model of the transition from the sleep state to the active state (equations (2.9)–(2.12)) on the basis of the following assumptions about a wireless sensor network.

**Figure 7.**
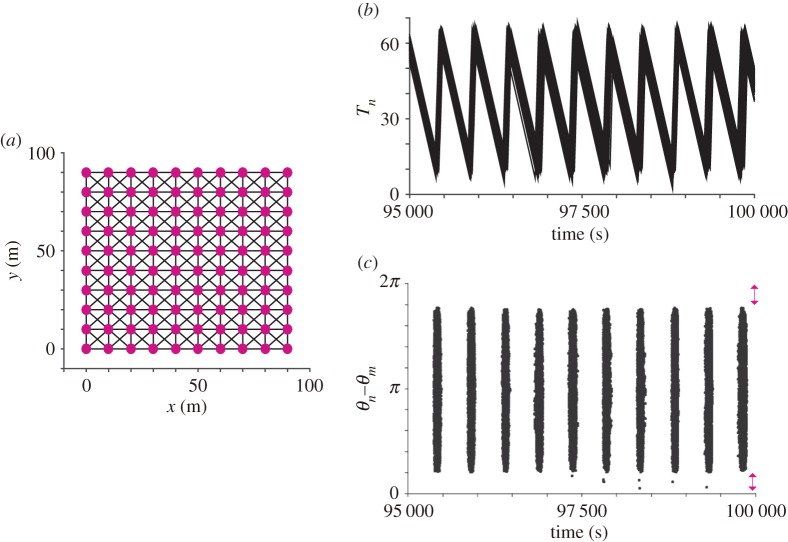
Numerical simulation on the application of our mathematical model to the control of a wireless sensor network. (*a*) Spatial structure of a wireless sensor network with 100 nodes. Each circle represents a node, and each line represents a pair of nodes that can interact with each other. Here, we assume the situation that 100 nodes were deployed on a two-dimensional square lattice and interact with a Moore neighbourhood. (*b*) Time-series data of *T*_*n*_. (*c*) Time-series data of the phase difference among neighbouring nodes. It is demonstrated that the nodes almost synchronize the timing of the transition between the active state and the sleep state (figure 7*b*) while avoiding in-phase synchronization with their neighbours (figure 7*c*).

— *Network structure*: In a wireless sensor network, the number of sensor nodes varies considerably from tens to thousands depending on its purpose [17,18]. As a representative case of a medium-scale network, we assume that 100 nodes are distributed on a two-dimensional square lattice at intervals of 10 m and interact with a Moore neighbourhood positioned within the range of $r_{0}=\sqrt{2}\times 10\;\text{m}$. The network structure assumed here is shown in figure 7*a*.
— *Deterministic models*: For practical use, each sensor node needs to quickly avoid a packet collision with its neighbours [17,18]. A packet collision is defined as the state in which two nodes send a data packet almost at the same time; such a state can be described as the in-phase synchronization of two oscillators (i.e. *θ*_*n*_ − *θ*_*m*_ ∼ 0 or 2*π*) in the framework of a phase oscillator model. To quickly avoid the in-phase synchronization, we assume the following interaction term in equation (2.1):
  3.1 $$\Gamma_{nm}(\theta_{n}-\theta_{m})=-\frac{K_{nm}}{\pi }(\theta_{n}-\theta_{m}-\pi ).$$
  For the model of frog choruses, we used the sinusoidal interaction term of equation (2.13). From the viewpoint of practical application to a wireless sensor network, there is a problem with the sinusoidal term: namely, *Γ*_*nm*_(*θ*_*n*_ − *θ*_*m*_) approaches 0 near the in-phase synchronization (see electronic supplementary material, figure S2A), indicating that it takes a long time for sensor nodes to avoid a packet collision once it occurs. By contrast, we use the linear term of equation (3.1) for the control of a wireless sensor network. The point is that this linear term takes a large positive value of *K*_*nm*_ at *θ*_*n*_ − *θ*_*m*_ = 0 and a large negative value of − *K*_*nm*_ at 2*π* (see electronic supplementary material, figure S2B). This discontinuity at *θ*_*n*_ − *θ*_*m*_ = 0 and 2*π* makes the in-phase synchronization very unstable, allowing the nodes to quickly avoid the in-phase synchronization with their neighbours. Here, we fix the coupling coefficient as *K*_*nm*_ = 1 that is larger than the value assumed in the case of frog choruses so as to achieve the quick avoidance of in-phase synchronization. We then fix the intrinsic angular velocity of the phase oscillator model as *ω* = 2*π*; this assumes a simple situation in which an active node sends a data packet at the interval of almost 2*π*/*ω* = 1.0 s. The other parameter of the deterministic models is assumed to be governed by the same relationship as in the case of frog choruses (i.e. *α* = 0.12 × *ω*/2*π*).
— *Stochastic models*: A wireless sensor network is required to collect data for a long time by reducing its power consumption [17,18]. To achieve this requirement, we fix the parameters of our stochastic models as *E*_max_ = 50 000 and Δ*T* = 60; these values imply that a sensor node has a high battery level (*E*_max_ = 50 000) at the initial condition and then cyclically switches into the sleep state after sending a data packet approximately 60 times (Δ*T* = 60). Along with this change, we increase the steepness of the logistic functions *G*_1_(*T*_*n*_) and *G*_2_(*T*_*n*_) (equations (2.8) and (2.10)) as *γ* = 1.5 in order to make the time scale of the transition more robust. The other parameters are fixed at the same values or are governed by the same relationship as in the case of frog choruses (i.e. *β* = 0.01, *p*_high_ = 0.80, *p*_low_ = 0.01 and *T*_max_ = 1.2 × Δ*T*).

To perform numerical simulation, we fix the interval between the updates of the node’s state as Δ*t*_update_ = 5.0 s that is much longer than the interval of data transmission (i.e. 2*π*/*ω* = 1.0 s).

### 3.3. Numerical simulation

To examine the efficacy of the proposed model as the control method of a wireless sensor network, we perform numerical simulation by using equations (2.1)–(2.12) with the parameter values that are fixed as explained in §3.2. In regard to the initial conditions, the state *s*_*n*_, the physical fatigue *T*_*n*_ and the phase *θ*_*n*_ are randomized while the energy *E*_*n*_ is fixed at the maximum value *E*_max_. In addition, the phase *θ*_*n*_ is randomized every time the *n*th node switches its state from the sleep state to the active state, in the same way as with frog choruses. Figure 7*b* shows the time-series data of *T*_*n*_, which demonstrates that the sensor nodes collectively and cyclically switch between the active state and the sleep state. Given that each node can reduce its power consumption during the sleep state, this cyclic and collective feature is useful for prolonging the network lifetime while temporally increasing network connectivity. Figure 7*c* shows the time-series data of the phase difference. In this simulation, we calculate the phase difference only for a Moore neighbourhood positioned within the interaction range *r*_*nm*_ ≤ *r*_0_ so as to detect the occurrence of a packet collision among neighbouring nodes. It is demonstrated that the phase difference is not distributed around the in-phase synchronization of *θ*_*n*_ − *θ*_*m*_ ∼ 0 or 2*π*: namely, the nodes succeed in avoiding a packet collision with their neighbours.

**Figure 8.**
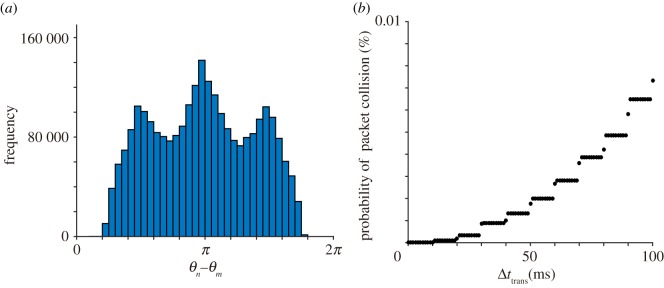
Numerical simulation on the avoidance of a packet collision. (*a*) Histogram of the phase difference among neighbouring nodes. This histogram demonstrates that the nodes avoid a packet collision (i.e. in-phase synchronization) with their neighbours. (*b*) The relationship between Δ*t*_trans_ and the probability of a packet collision. Here, the parameter Δ*t*_trans_ represents the duration of data transmission.

To quantitatively examine the performance of the proposed model, we carry out further simulation. As for the avoidance of a packet collision, we first calculate the histogram of the phase difference among a Moore neighbourhood from the time-series data between *t* = 0 and 100 000. The histogram demonstrates that the in-phase synchronization is robustly avoided by the proposed model (figure 8*a*). In the actual situation of a wireless sensor network, it typically takes around tens of milliseconds for each node to complete sending a data packet [34]. Based on this feature, we define the duration of a data transmission as a positive parameter Δ*t*_trans_, and then determine that a packet collision occurs every time two neighbouring nodes send a data packet within Δ*t*_trans_. Figure 8*b* shows the relationship between Δ*t*_trans_ and the probability of a packet collision, demonstrating that the probability is less than 0.01% in the range of 0 < Δ*t*_trans_ < 100 ms.

**Figure 9.**
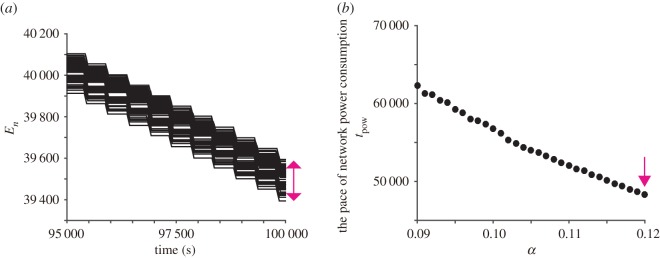
Numerical simulation on network power consumption. (*a*) Time-series data of *E*_*n*_. Because the battery level of each node is constrained as *E*_*n*_ = 50 000 at *t* = 0, the variance of the power consumption is approximately 2% at *t* = 100 000. (*b*) The relationship between *α* and *t*_pow_. Here, *α* represents the recovery rate of *T*_*n*_ during the sleep state (equation (2.5)) while *t*_pow_ represents the pace of network power consumption. A pink arrow depicts the parameter value assumed in the simulation of figure 7.

Next, we examine the feature of network power consumption over a long time scale. Figure 9*a* shows the time-series data of *E*_*n*_ after 95 000 s have passed from the start of the simulation. It is demonstrated that the variance of *E*_*n*_ is approximately 200 at *t* = 100 000. Because the initial condition is constrained at *E*_*n*_ = 50 000, this variance corresponds to about 2% of the total power consumption of respective nodes. To show the performance of power saving over a long time scale, we then examine how the pace of network power consumption is affected by the parameters of the proposed model. Especially, the parameter *α* affects the duty ratio of each node (i.e. the ratio of the durations of the sleep state and the active state) because this parameter represents the recovery rate of *T*_*n*_ during the sleep state (see equation (2.5)). We then define the pace of network power consumption *t*_pow_ as the time in which the remaining energy of all the nodes becomes less than 90% of *E*_max_, and examine the relationship between *t*_pow_ and *α*. Figure 9*b* demonstrates that *t*_pow_ takes a larger value when *α* takes a smaller value, indicating that we can control the network lifetime by the parameter *α*. The dependence of *t*_pow_ on the other parameters is partially shown in electronic supplementary material, figure S4 although those parameters do not strongly affect the value of *t*_pow_ compared to the parameter *α*.

## 4. Conclusion and discussion

In this study, we investigate the behavioural mechanisms of frog choruses from the viewpoints of mathematical modelling and its application. Our empirical data demonstrate that male Japanese tree frogs avoid call overlaps with their neighbours while collectively switching between the calling state and the silent state, which is consistent with the observations on various species of frogs [8–16]. We reproduce these features by using a hybrid dynamical model in which male frogs stochastically switch their states depending on the internal conditions of respective frogs as well as the interactions among them. Next, the mathematical model is applied to the control of a wireless sensor network. Numerical simulation shows that the proposed model allows sensor nodes to avoid a packet collision over a short time scale while cyclically and collectively switching their states over a long time scale. We consider that these features are useful for the autonomous distributed control of a wireless sensor network (see the last three paragraphs of this section for details).

From the viewpoint of mathematical modelling on frog choruses, it remains as an important future problem to extend the proposed model to a system of many frogs that can be observed in natural environment [8,9,27]. In this study, we focus on the chorus of just two frogs or three frogs; the reason of this restriction is the difference in the data size between our indoor experiments and field observations. Namely, the data of our indoor experiments consist of the audio recordings of 16 h (4 h × 4 datasets as described in [12]) while the data of our field observations consist of the video recordings of just 1.25 h (15 min × 5 datasets as described in [27]). Therefore, the datasets of our field observations are currently much smaller than those of the indoor experiments; we then focus on the system of two frogs or three frogs in this study to use the larger datasets of the indoor experiments. On the other hand, we previously observed a specific temporal structure (i.e. two-cluster synchronization) in natural choruses of male Japanese tree frogs, and reproduce the structure by using a phase oscillator model in which the interaction decays depending on the distance among male frogs [27]. Given that network topology of the frog choruses (i.e. the spatial distributions of male frogs) can change every night, it would be important to theoretically analyse the relationship between the temporal structure and the network topology over multiple time scales by extending the proposed model to a system of many frogs.

In this study, we have proposed a novel model for the control of a wireless sensor network in which each sensor node autonomously controls the timing of data transmission by using only local network information (§§3.1 and 3.2). The main features of this model are summarized as follows: (1) neighbouring nodes can avoid a packet collision over a short time scale by alternating the timing of data transmission (figures 7*c* and 8) and (2) all the nodes collectively switch their states over a long time scale, establishing high network connectivity while reducing network power consumption (figures 7*b* and 9). Here, we discuss the efficacy and novelty of these features from the viewpoint of the application to a wireless sensor network.

As for the first feature over a short time scale, our simulation demonstrates that the probability of a packet collision is less than 0.01% in the range of 0 < *t*_trans_ < 100 ms. This performance needs to be compared with the performance of alternative methods such as CSMA/CA [35], DESYNC [36] and another method inspired by frog choruses [19], although those methods do not realize the collective transition between the active state and the sleep state over a long time scale. In addition, further analysis is required to examine the detailed temporal structure of the phase difference among neighbouring nodes. In particular, we have confirmed that a packet collision can be avoided among neighbouring nodes (figure 8), but could not find a specific temporal structure of the phase difference (see electronic supplementary material, figure S3, which represents the enlargement of figure 7*c*). Our previous study using a mathematical model showed that anti-phase synchronization of two oscillators produces a complicated temporal structure of the phase difference in a system with many oscillators (i.e. two-cluster synchronization and also wavy anti-phase synchronization with various wavenumbers) even when we assume a very simple spatial structure of a ring [27]. Given that we assume a more complicated spatial structure in this study (i.e. a two-dimensional square lattice in which each node interacts with a Moore neighbourhood), other types of temporal structures are likely to emerge. It is also an important future problem to change the topology of a wireless sensor network and then examine the relationship with the temporal structure of the phase difference because the network topology can vary a lot depending on a practical application of a wireless sensor network in general. In regard to this point, Belykh *et al.* showed that network topology affects the coupling strength necessary to achieve complete in-phase synchronization of a network in a system of coupled oscillators that attempt to synchronize at in-phase with other oscillators [37]; such a viewpoint on the relationship between the temporal structure and the network topology is important for examining the performance of the proposed model on the avoidance of a packet collision in details.

As for the second feature over a long time scale, we consider that the proposed model provides a novel approach on the control of a wireless sensor network. The point is that the proposed model realizes the cyclic and collective transition among the nodes on the basis of their excitatory interaction (equation (2.12)). To our knowledge, similar methods have not been proposed yet, although some studies have achieved the reduction in energy consumption through an inhibitory interaction among nodes [19]. Future problems include the detailed analysis of the performance of energy saving over a long time scale by varying the network topology.

## Supplementary Material

SI20181125.pdf

## Data accessibility

All data essential to evaluate the conclusions of this study are present in the manuscript.
