# Supplementary material for: Mathematical modelling and application of frog choruses as an autonomous distributed communication system
Source: R Soc Open Sci. 2019 Jan 9;6(1):181117. doi: 10.1098/rsos.181117 (PMC6366160; doi:10.1098/rsos.181117)
Supplement: SI20181125.pdf [file rsos181117supp1.pdf]

Supplementary Information: Mathematical Modelling and Application of Frog Choruses as an Autonomous Distributed Communication System

Ikkyu Aihara[1,*], Daichi Kominami[2], Yasuharu Hirano[3], Masayuki Murata[3]

1 Graduate School of Systems and Information Engineering, University of Tsukuba, Ibaraki 305-8573, Japan

2 Graduate School of Economics, Osaka University, Osaka 560-0043, Japan

3 Graduate School of Information Science and Technology, Osaka University, Osaka 565-0871, Japan

∗ **E-mail: aihara@cs.tsukuba.ac.jp**

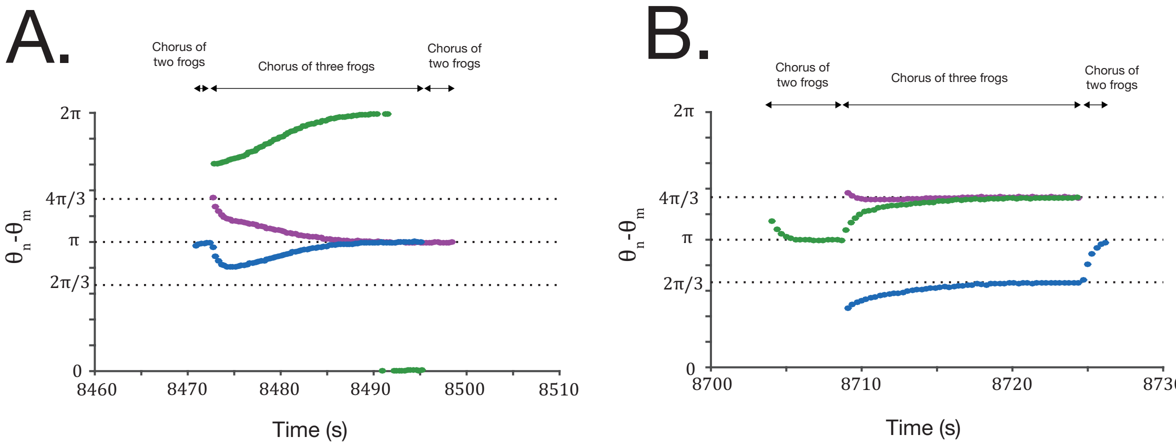

Figure S1. Numerical simulation of our mathematical model on frog choruses. (A) Clustered anti-phase synchronization of three frogs. (B) Tri-phase synchronization of three frogs. These figures correspond to the enlargement of the bottom panel of Figure 4B. In the chorus of three frogs, the phase differences converge to a set of $(0, \pi)$ in Figure S1A and converge to a set of $(2\pi/3, 4\pi/3)$ in Figure S1B; this result means that clustered anti-phase synchronization and tri-phase synchronization occur in the proposed model. Note that, before and after the chorus of three frogs, there are short periods in which only two frogs make a chorus; in those period, a phase difference converges to π (i.e., anti-phase synchronization of two frogs is realized).

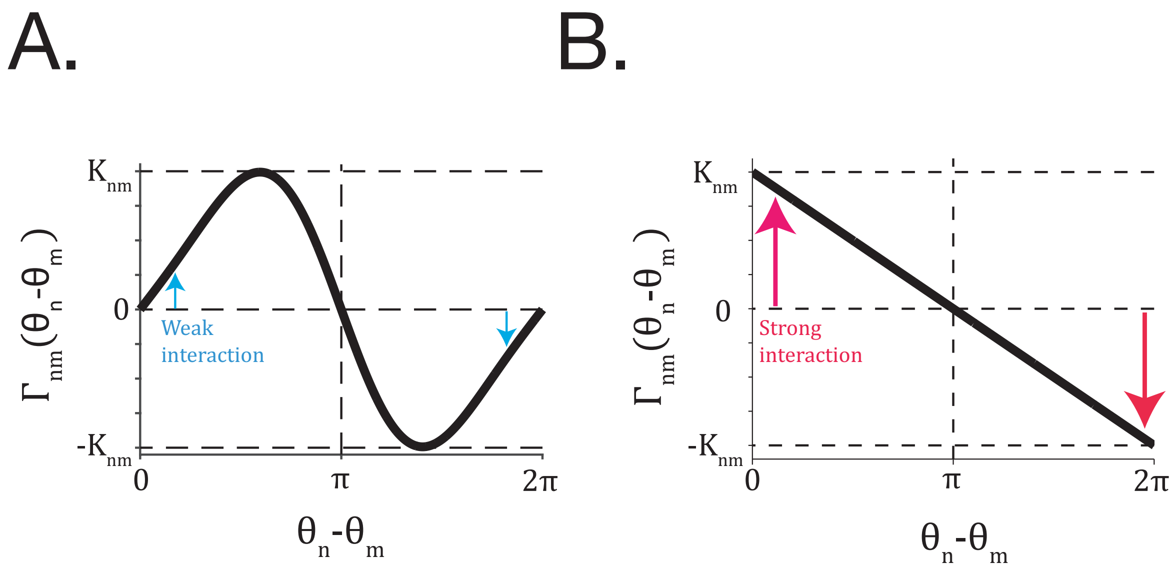

Figure S2. The purpose of the usage of a linear interaction term (Equation (3.1)) for the control of a wireless sensor network. (A) A sinusoidal interaction term with the second-order component ($k = 0.18$). The interaction is almost zero near the in-phase synchronization. (B) A linear interaction term. This term has a large positive value at $\theta_n - \theta_m = 0$ and a large negative value at 2π, allowing each node to quickly avoid in-phase synchronization with their neighbors.

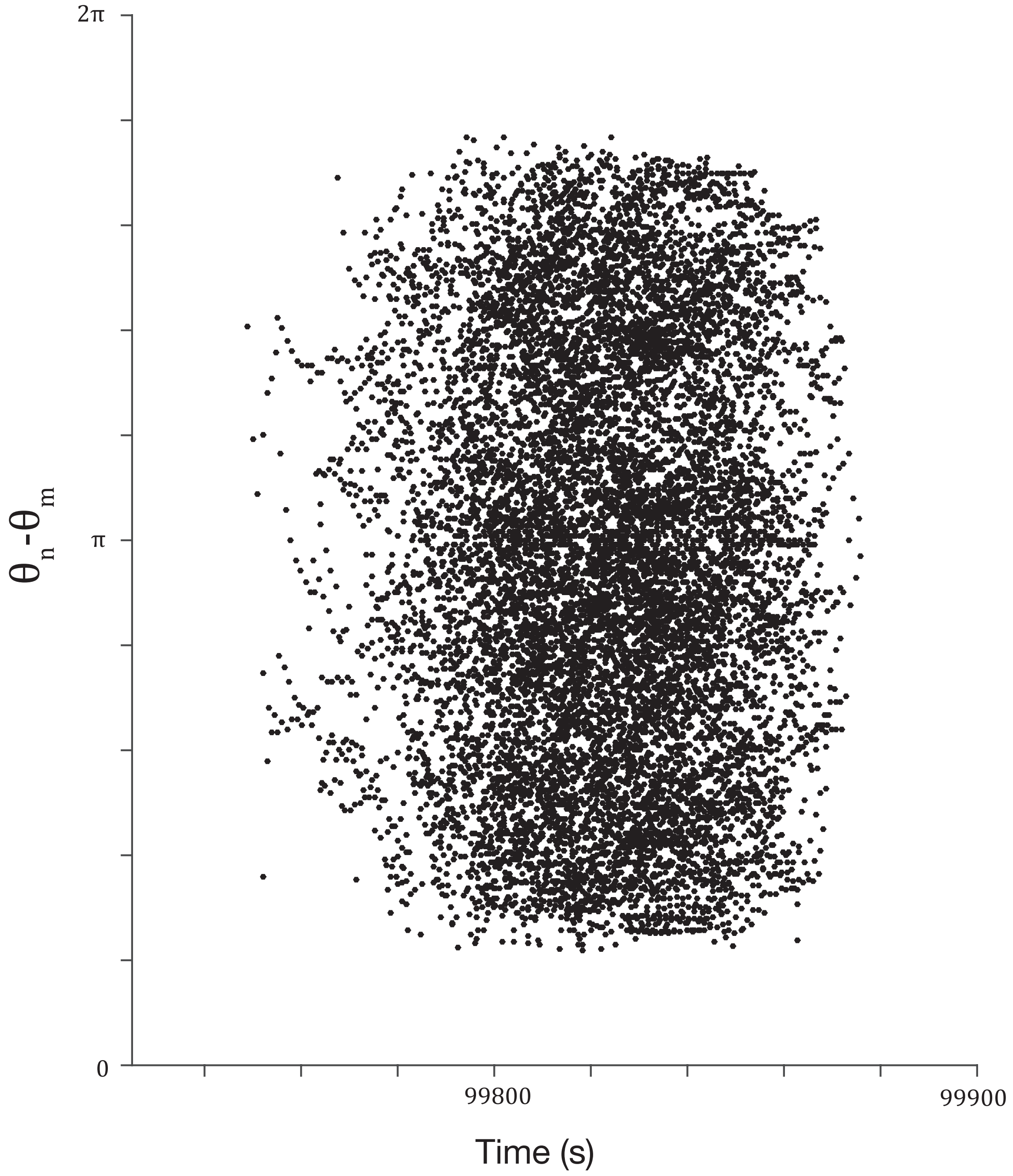

Figure S3. Numerical simulation of our mathematical model on a wireless sensor network. This figure corresponds to the enlargement of Figure 7C in the main manuscript, showing the time series data of the phase difference among neighboring nodes. It is indicated that a very complicated temporal pattern occurs in the phase dynamics.

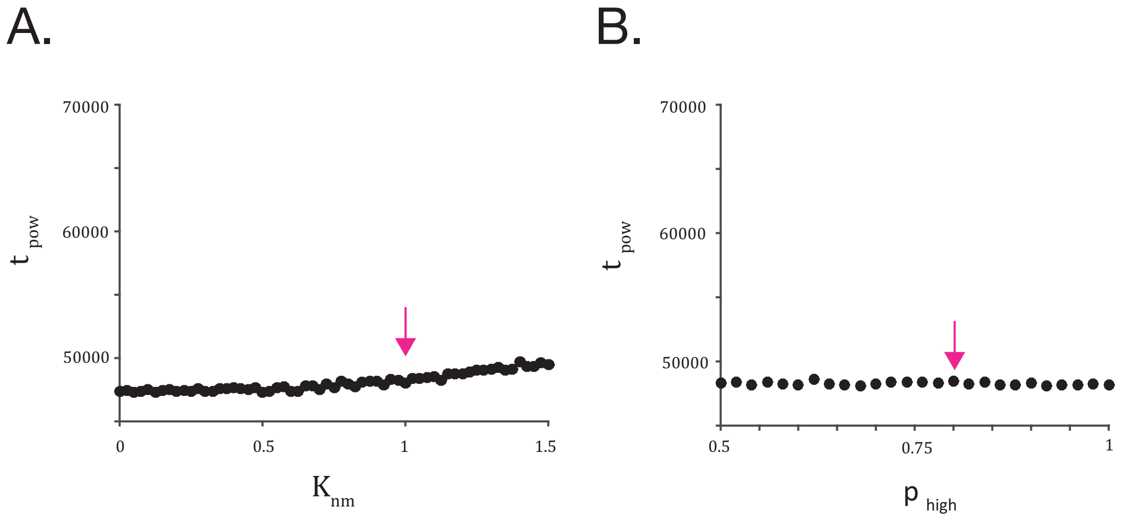

Figure S4. Numerical simulation of our mathematical model on a wireless sensor network. (A) Relationship between the pace of power consumption t_{pow} and the parameter K. (B) Relationship between the pace of power consumption t_{pow} and the parameter p_{high}. The range of the vertical axe is set at the same value with Figure 9B. The parameter value used in the simulation of Figure 7 is depicted by a pink arrow in each figure. These two parameters do not strongly affect the value of t_{pow} compared to the parameter α (see Figure 9B).
